# A Study on the Identification of Five Arboviruses from Hematophagous Mosquitoes and Midges Captured in Some Parts of Northern Turkey

**Published:** 2019-06-24

**Authors:** Emre Ozan, Harun Albayrak, Semra Gumusova, Cenk S. Bolukbas, Mithat Kurt, Gokmen Z. Pekmezci, Yunus E. Beyhan, Hamza Kadi, Selma Kaya, Ismail Aydin, Zafer Yazici

**Affiliations:** 1 Veterinary Control Institut, Ministry of Food Agriculture and Livestock, Samsun, Turkey; 2 Department of Virology, Faculty of Veterinary Medicine, Ondokuz Mayis University, Samsun, Turkey; 3 Department of Parasitology, Faculty of Veterinary Medicine Ondokuz Mayıs University, Samsun, Turkey; 4 Department of Aquatic Animal Diseases, Faculty of Veterinary Medicine, Ondokuz Mayis University, Samsun, Turkey; 5 Department of Parasitology, Faculty of Medicine, Yuzuncu Yil University, Van, Turkey

**Keywords:** Mosquito, Midge, Identification, Real-time, Arbovirus

## Abstract

**Background:**

Whether zoonotic or not, arboviral infections are continuing to be a major threat to human health as well as the livestock industry all around the world. This project presented the results of the identification study on five arboviruses, including West Nile virus (WNV), Bovine ephemeral fever virus, Akabane virus, Bluetongue virus, and Epizootic hemorrhagic disease virus, in mosquitos and midges from eight provinces of the Black Sea Region.

**Methods:**

During 2011 and 2012, 3193 mosquitoes were captured around natural streams, rivers, lakes, and ponds using dry-baited miniature light-traps. Identification studies were concluded by employing molecular methods.

**Results:**

According to the morphological identification, blood-sucking mosquitoes and biting-midges belonged to *Aedes* (44.69%), *Anophele*s (28.34%), *Culex* (22.14%) and Culicoides (4.83%) species. Overall, 146 pools were made up of captured mosquitos and midges. None of the five viruses were directly identified by mosquitoes.

**Conclusion:**

Mosquitoes and midges have got a crucial role in the transmission of arboviruses. The risk of occurrence for the investigated arboviruses will continue depending upon many factors including the presence of these viruses in Turkey and its neighboring countries, uncontrolled livestock movements, global warming and climate changes.

## Introduction

Viruses transmitted by arthropod vectors can either be zoonotic or non-zoonotic. Currently, more than 100 species of arbovirus are categorized as zoonoses due to their transmissibility from arthropods to human (1). Most arboviruses belong to the virus families Flaviviridae, Togaviridae, Reoviridae as well as both Peribunyaviridae and Phenuiviridae within the order Bunyavirales, formerly known as Bunyaviridae (1–3). Mosquitoes and ticks are the main vectors for the majority of arboviruses (1, 4). There are approximately 300 species of mosquito and 116 species of ticks known to be vectors of arboviruses (5, 6). The vast majority of mosquitos are *Aedes* and *Culex* species, with 115 and 105 species respectively (6). In addition, 25 midge species, mainly *Culicoides*, and Sandflies, Lasiohelea blackflies, stink-bugs, lice, mite, might have played a role in transmitting arboviruses (5). Even though certain viruses are transmitted by specific vectors, some arboviruses, for example, West Nile virus (WNV), can be transmitted additionally by ticks and other arthropods (1).

Infection with certain arboviruses can result in serious symptoms, including high fever, haemorrhage, meningitis, encephalitis and death (1). Moreover, in animals, abortions, stillbirth, congenital anomalies arthrogryposis-hydranencephaly are also seen in as a result of infection by certain arboviruses, for example, Bluetongue virus (BTV), and Akabane virus (AKAV). Equally, some arboviral infections are asymptomatic and the patients recover from infection after a few weeks.

WNV, dengue virus (DENV) and Chikungunya virus (CHIKV) are the best known and globally distributed human arboviruses (1, 7). BTV, AKAV and Bovine ephemeral fever virus (BEFV) are important, non-zoonotic arboviruses of animals. In particular, large epidemics of BTV-serotype 8 in EU countries including Holland, Czech Republic, Belgium, Sweden, the UK, and Switzerland emerged between 2006–2008 and caused major economic losses in all of these countries (8).

In Turkey, the presence of WNV, BTV, AKAV, BEFV and Epizootic hemorrhagic disease virus (EHDV) has been reported following serological studies (9–12). Furthermore, a limited number of studies have been conducted to identify arboviruses from invertebrate vectors (7).

The spread of arboviruses is influenced by many factors, such as increasing international travel, globalization of trade and climate change. Whether symptomatic or not, arboviral infections may cause a large social and economic burden for societies (1). The detection of arboviruses in vectors is important for public health and the livestock industry and allows a lot of detailed information to be gathered about seasonal arbovirus circulation, helping to create time-dependent preventive measures.

As the geographical bridge between Europe and Asia, Turkey has a rich capacity as for natural and ecological conditions that provide a suitable habitat for arthropod populations, thus paving the way for the emergence of new arthropod-borne viruses. At the same time, Turkey is also at risk due to the known presence of these viruses in neighboring countries such as Iran, Iraq, Syria, the Balkan and Blacksea states.

The aim of this study was to identify five arboviruses from field trapped mosquitos and midges at different provinces in Northern Turkey. Captured vectors were examined by employing the real-time RT-PCR and RT-PCR.

## Materials and Methods

### Samples

**Fig. 1.**
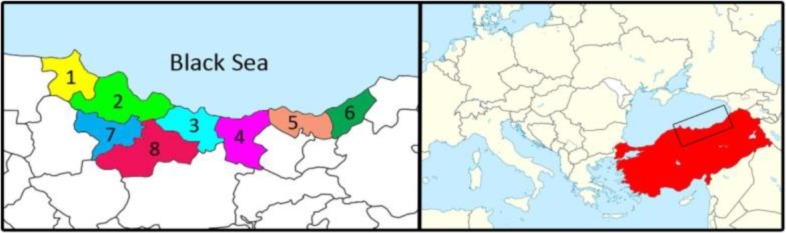
A map showing the provinces targetted in htis study for the capture of mosquitoes and biting-midges during 2011 and 2012. Provinces that indicated with numbers are 1: Sinop, 2: Samsun 3: Ordu, 4: Giresun, 5: Trabzon, 6: Rize, 7: Amasya, 8: Tokat

During summer months of 2011 and 2012, eight provinces of Northern Turkey (Fig. 1) were chosen to collect mosquito and midge samples. In the sampling period, two significant points were taken into account for the installation of traps: (i) areas of sampling where mosquito and midges were particularly abundant, such as cowsheds and sheepfolds, near or close to lakes, ponds, dam-lakes, natural streams and rivers, were chosen in the immediate vicinity of least 3 residential area in each province. (ii) Mosquitos and midges were collected on days that were without rain and wind during the night between 6pm and 12pm. Mosquitos and midges were caught in glass containers containing water and then were transported immediately to the laboratory under cold chain.

Mosquitoes and midges were separated under the stereomiscroscope in order to morphologically identify species (13, 14). Overall, 146 pools from 3193 mosquitos and midges were made up according to each capture province and capture time, and were stored at −80 °C until use. The vast majority of them were *Aedes* spp. corresponding to 1427 (44.69%) mosquitoes. Furthermore, we identified the numbers of *Anopheles* spp., *Culex* spp. and *Culicoides* spp. as 905 (28.34%), 707 (22.14%) and 154 (4.83%), respectively (Table 1).

**Table 1.** The numbers of captured insect species according to provinces where the traps were installed

**Longitude (°E)**	**Latitute (°N)**	**Trapping Provinces**	**Captured Mosquitos and Midges**			

			***Aedes***	***Anopheles***	***Culex***	***Culicoides***
35.09	42.01	Sinop	19	540	158	27
36.20	41.17	Samsun	1372	195	168	44
36.43	40.19	Amasya	3	20	46	20
35.50	40.40	Tokat	23	136	181	35
37.53	41.00	Ordu	3	3	82	26
38.24	40.55	Giresun	-	4	35	2
39.43	41.00	Trabzon	5	4	60	-
40.31	41.02	Rize	2	3	7	-
**Total**			**1427**	**905**	**707**	**154**

### Homogenization of mosquitoes and midges

Insect pools were transferred into magnalyser green bead tubes (Roche Germany). 1ml of PBS (Gibco) was then added to each tube. Pools were homogenized using a 1.4mm magnalyser ceramic bead by shaking MagNa Lyser (Roche, Germany). Following homogenization, the unsoluable particulates were pelleted by centrifugation at 4 °C, 3000rpm for 15min.

The supernatant from each pool was then transferred into a new tube and used for nucleic acid purification using the total RNA extraction kit (Qiagen, Spain) according to the manucfacturer’s instructions.

### RNA extractions and RT-PCR tests

Positive controls of viral RNA used for PCR tests were kindly provided by University of Texas Medical Branch (Galveston, USA) for WNV, Pendik Veterinary Control Institute (Istanbul, Turkey) for BTV, Veterinary Diagnostic Technology Inc (USA) for EHDV, Veterinary Control Institute (Samsun, Turkey) for AKAV and Department of Virology, Faculty of Veterinary Medicine, University of Selcuk (Konya, Turkey) for BEFV. Detailed information regarding primers and Taqman probes labelled FAM-TAMRA are given in Table 2.

**Table 2.** Primers and Probes sequences that employ for this research

**Primer/Probes**	**Sequences(5′-3′)**	**Position**
WN3 NC-F	CAGACCACGCTACGGCG	10,668–10,684
WN3 NC-R	CTAGGGCCGCGTGGG	10,770–10,756
WN3 NC-P	TCTGCGGAGAGTGCAGTCTGCGAT	10,691–10,714
BTVrsa 291–311F	GCGTTCGAAGTTTACATCAAT	291–311
BTVrsa 387–357R	CAGTCATCTCTCTAGACACTCTATAATTACG	387–357
BTVuni 291–311F	GCTTTTGAGGTGTACGTGAAC	291–311
BTVuni 381–357R	TCTCCCTTGAAACTCTATAATTACG	381–357
BTV 341–320P	CGGATCAAGTTCACTCCACGGT	341–320
BTV 346–323P	TCCTCCGGATCAAGTTCACTCCAC	346–323
EHD 1/2F	GCGTTGGATATATTGGACAAAGC	165–187
EHD 1/2R	GCATACGAAGCATAAGCAACCTT	275–253
EHD 1/2P	TCAAATCAAACGGGCGCAACTATGG	192–216
EHD 3F	AGCCCTCGACATTCTGGATAAG	164–185
EHD 3R	CGCGACTTTCTCCACTTTTTG	259–239
EHD 3P	CAAATCAAACTGGTGCCACGATGGC	206–230
EHD 4F	AAGTTGCCCTCGATATCCTAGATAAG	160–185
EHD 4R	GTAAGCGACCTTTTCTACCTTTTGA	266–242
EHD 4P	CAATGTCTAACCAAACAGGCGCTACAATGG	199–228
EHD 5F	GCTGGATATACTCGACAAAGCAATG	167–191
EHD 5R	ACGCGACCTTTTCTACTTTTTGA	262–240
EHD 6F	GAGTCGCGCTGGATATACTC	160–179
EHD 6R	GCATATGATGCATACGCGACCTTT	276–253
EHD 7F	CAAAAGTTGCCCTTGACATTTTAG	157–180
EHD 7R	CATATGCTACTTTTTCTACCTTCTGC	266–243
EHD 8F	CCAAAAGTCGCCCTTGATATTT	156–177
EHD 8R	CATATGCCACTTTTTCTACCTTCTGC	268–243
AK151F	GCTAGAGTCTTCTTCCTCAACCAGAA	151
AK231R	AAAAGTAAGATCGACACTTGGTTGTG	231
AK182P	CCAAGATGGTCTTACATAAG	182
BEF346 F	TATTACCCTCCTGCCGGATGCTTT	346–369
BEF1155 R	AGGTCTGTATTCGCACCAAGCTCT	1155-1132

Viral RNAs of WNV, BTV, EHDV and AKAV was detected in 96 well plates using a Light Cycler 2.0 real-time PCR systems (Roche, Germany). The results were assessed using the LightCycler Software 4.05 (Roche, Germany). Arktik thermal cycler (Thermo Scientific, UK) were used for RT-PCR in order to detect BEFV RNA.

The concentration and purity of RNA were used by absorbance at 260–280nm wavelenghts using a UV spectrophotemeter (Helios Gamm, Thermo Spectronic, UK).

To perform a real-time RT-PCR for the detection of WNV-RNA in mosquito pools, specific primers and a probe (15) were used. The Taqman probe was labeled at the 5′ end with the reporter dye FAM and labeled at the 3′ end with quencher dye TAMRA. Briefly, the reaction mixture was made up in a total volume of 50µL containing 2µL sample RNA, 75mM Tris-HCl (25 °C, pH 8.8), 20mM (NH_4_)_2_ SO_4_, 6mM MgCl_2_, 0.2mM of dNTP mix, 50 pmol of primers 10pmol of prob, 1µL of Triton X-100, 5U RNase inhibitor, 100U Moloney murine leukemia virus reverse transcriptase and 2,5U Taq DNA polimerase (Thermo Scientific, UK) and water. The mixture was cycled as follow: 1 cycle 30min at 42 °C for reverse transcription, 1 cycle of 2min at 94 °C, 40 cycles of 30sec at 94 °C, 30sec at 55 °C and 20sec at 72 °C and 1 cycle of 30sec at 40 °C.

To investigate BTV-RNA in pools, the real-time RT-PCR method (16) was carried out using the QIAamp Viral RNA Mini Kit (Qiagen, Spain) according to the manufacturer’s instructions. RNA was eluted in 50μl nuclease-free water and then stored at −20 °C. For RNA sample denaturation prior to the initial RT step, heat denaturation of RNA was evaluated by heating 6μl samples at 98 °C for 5 min followed by rapid cooling on ice. The reaction mixture contained, 20pmol of each primer, 5pmol of prob, 1.25mM MgCl_2_, 0,4mM dNTPs, 0.8µL of enzyme mix, 4µL of 5X RT-PCR buffer, 3µL denatured RNA and water to a final volume of 20µL.The mixture was cycled in using following procedure: After 1 cycle of 30min at 50 °C for reverse transcription, 1cyle of 15min at 95 °C for denaturation, 45 cycles of 15sec at 95 °C and 1min at 60 °C. Test was evaluated at the end of 30s at 40 °C.

To investigate AKAV-RNA in pools, we used touch-down rRT-PCR with some modifications (17). The reaction mixture was made up in a total volume of 50µL containing 3µL sample RNA, 75mM Tris-HCl (25 °C, pH 8.8), 20mM (NH_4_)_2_SO_4_, 6mM MgCl_2_, 0.2mM dNTP Mix, 20pmol of primers 10pmol of prob, 1µL of Triton X-100, 5U RNase inhibitor, 100U Moloney murine leukemia virus reverse transcriptase and 2.5U Taq DNA polymerase (Thermo Scientific, UK). Cycling conditions included: RNA was reverse transcribed at 42 °C for 30min and was followed by an inactivation step at 94 °C for 2min). After reverse-transcription and inactivation steps, 10 cycles of 15sec at 95 °C, 60sec at 60 °C (decreased by 2 °C per cycle so that it reached 50 °C after 10 cycles) and 15min at 72 °C were carried out. Finally, a further 35 cycles of 15sec at 95 °C, 1min at 50 °C and 15sec at 72 °C and 40 °C for 2min followed.

To investigate EHDV-RNA in pools, the mixture was prepared in a total volume of 20 µL containing 3µL sample RNA, 4µL of 5X master mix, 1.25mM MgCl_2_, 0.5mM dNTPs, 20pmol of primers, 5 pmol of probe, 1µL of enzyme mix (Qiagen, Spain) and water. Cycling conditions of the reaction were as follows: 1 cycle of 30min at 50 °C for reverse transcription step, 15min at 95 °C for inactivation and denaturation step, and 40 cycles of 10sec at 95 °C, 1min at 55 °C, 30 sec at 72 °C and finally incubation 30sec at 40 °C. Seven primer pairs and 3 probes were used to investigate all of the EHD serotypes. EHDV-1 and 2 were tested using the same primer pair. The different probes were used for EHDV 3 and 4 while the same probes were using for EHDV-1, 2, 5, 6, 7 and 8 (18).

BEFV-RNA in pools were tested by using one-step reverse transcription PCR method. Specific primers with the Qiagen OneStep RT-PCR kit were used for ampflying a 809bp DNA fragment (19). The RT-PCR mixture was prepared in a total volume of 50µL containing 5µL sample RNA, 10µL of 5X mastermix, 0.2 mM of dNTP mix, 10pmol of primers, 10U RNase inhibitor and 2µL of enzyme mix and water. Amplification was conducted using thermal cycler according to the reaction conditions as follow: following reverse-transcription at 50 °C for 30min, 1 cycle of 5min at 95 °C, then 35 cycles of 30sec at 95 °C, 1min at 56 °C and 1min at 72 °C. The amplication process was terminated after a final extension of 10min at 72 °C. 5µL of PCR products were visualized on a 1.5% agarose gel stained ethidium bromide.

## Results

**Fig. 2.**
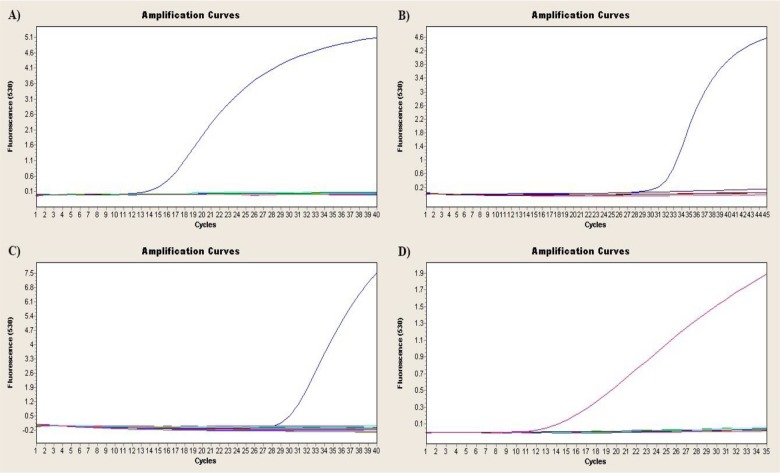
The amplication results of four arbovirus including WNA, BTV, EHDV and AKAV by a real time RT-PCR test. Curves indicated the real time PCR plots of positive controls. A:WNV, B:BTV, C: EHDV, and D:AKAV. A detectable fluorescence signals above the threshold for positive controls of four viruses occurred at 12 cycles for WNV, 28 cycles for BTV, 28 cycles for EHDV and 11 cycles for AKAV.As it seen in all figures, no positive pools for 4 viruses could be determined

Hematophagous mosquitos and biting midges were caught using dry-baited miniature light-traps located near to natural streams, rivers, lakes and ponds. Following morphological identification under the stereomicroscope, 146 pools were made, representing 3193 mosquitos and biting midges. At the end of morphological identification, overall proportions of hematophagous mosquitos and biting-midges were found to be 95.17% and 4.83%, respectively. Captured mosquitos were found to belong to *Aedes* (44.69%), *Anopheles* (28.34%) and *Culex* (22.14%) species. Midges were identified as *Culicoides* (4.83%),

The amplication results of four arbovirus including WNA, BTV, EHDV and AKAV by a real time RT-PCR test were shown in Fig. 2. Positive controls for 4 viruses worked very well and gave a good amplification earlier. A detectable fluorescence signals above the threshold for positive controls occurred at 12 cycles for WNV, 28 cycles for BTV, 28 cycles for EHDV and 11 cycles for AKAV. No viral RNA for these viruses could be identified by employing real-time RT-PCR in any of the insect pools. Similarly, BEFV-RNA was not detected using one-step RT-PCR in any of the mosquito pools.

In all of 8 provinces, none of five viruses could be determined during the sampling period.

## Discussion

Whether zoonotic or not, arboviral infections are continuing to be a major threat to human health as well as the livestock industry all around the world (1, 20). In the last decade, many outbreaks in humans and animals caused by arboviruses have been reported. Identification of arboviruses in vectors can provide us much information to help develop control strategies against them. Historically, there are only a few studies on arboviruses in vectors in Turkey, limited to the Flaviviridae and Togaviridae (7, 21). In one such study, 15.9% of collected mosquitos were reported as WNV positive (7). Our study was planned for five animal arboviruses, including WNV, BTV, EHDV, AKAV and BEFV to identify from their vectors. With the exception of WNV, all of the viruses are non-zootic. This study can be defined as the first compherensive identification study of mosquitoes and midges caught in the Middle and East Black Sea Region in Turkey.

During 2011–2012, 3190 mosquitoes and midges were collected using light traps in 8 provinces of the Central and East Blacksea region of Northern Anatolia, Turkey. According to the morphological identification results, the vast of majority of the overall vector population was mosquito (95.17%), comprising 44.69% *Aedes*, 28.34% *Anopheles* and 22.14% *Culex*, species known as vector for WNV, BEFV (22, 23). *Culicoides* midges, known as the important biological vector of BTV (8), EHDV (11) and AKAV (24), represented 4.83% of catches.

In Turkey, serologically the presence of WNV for the first time was reported in different animal species including horses (13.5%), dogs (37.7%), cattle (4%) and sheep (1%) (9). The first identification of WNV from haematophagous mosquitoes in Eastern Thrace territory of Turkey was carried out (7). In the previous years, a few identification studies on WNV had been reported in the Black Sea region and no WNV could be identified from insects and blood samples of various animal species such as horses and wild birds (25, 26). Moreover, the serological diagnosis of WNV could also not be possible in various animal species with the exception of goats that seropositivity rate was reported as 2.85% (27, 28). In our comprehensive study, although 80% of the captured insects were a specific vector for WNV, the identification of WNV was not possible using real-time RT-PCR. The lack of presence of WNV may be due to the absence of certain vector specific determinats of emergence, such as the seasonal activity, climate change, ecological and environmental conditions (7, 9).

BTV and EHDV both belong to the family Reoviriadae, and both viruses are transmitted between ruminant hosts by *Culicoides* biting midges. During the past decade, BTV outbreaks have occurred in the western part of Turkey (8, 29). An EHDV outbreak was also reported in Western Anatolia in 2007 (11). However, infections caused by these viruses in Northern Anatolia had not been seen before. On the other hand, there are serological data regarding both viruses showing the historical presence of these viruses. Seropositivity for EHDV had been reported in cattle as 1.33%, with evidence of BTV seropositive animals, including 11% for cattle, 3% for sheep and 4% for goat (25, 27–28, 30). In our study, no BTV-RNA and EHDV-RNA could be identified by using real-time RT-PCR. When scrutinized, these results could depend upon two main causes. First, historically neither disease has been clinically evident in this region until recently and, secondly, the proportion of *Culicoides* species was far fewer than other insects captured during sampling periods. Furthermore, livestock movements inside Turkey may confound some interpretations of serological data.

*Culicoides* is also the main vector for AKAV. Despite a serological prevalence of 22% in our study area (27), in neither mosquito nor midge pools could be not detected AKAV-RNA by real-time PCR. This result can be interperented similarly to BTV and EHDV.

In previous years, numerous BEFV outbreaks were reported in Turkey, primarily in South and Sout-East Anatolia. The climate of these regions is suitable for BEFV vectors. In addition, there are common borders with Syria and Iraq where the virus has been previously reported. The latest outbreak began in the same regions in 2012 and reached a case fatality rate (31, 32). In the Central and Eastern Blacksea region of Turkey, antibodies against BEFV were shown to be 13.5% (27). Despite the serological indications, clinical BEFV has not been reported. In the current study, we attempted to identify BEFV from captured insects using RT-PCR. At the end of the experiments, no BEFV-RNA could be found in all pools. According to our standpoint, there are a few unsuitable climatic conditions and vector ecology may represent important factors in determining whether or not infection occurs.

No viral RNAs of five arboviruses could be found in pools made up of captured various mosquitos and midges during this project. Infections caused by these five viruses were not observed clinically in the Central and East Blacksea region where the project was carried out. However, in the light of serological data obtained from previous studies, five viruses are nevertheless considered to circulate between animals and prospective vectors. Furthermore it is not ruled out that mosquitos and midges cannot be found in some locations of targeting regions or were never infected with five viruses.

Reservoir, vector, and climate are the most important aspects for the epidemiology of arboviruses. The climatic condition of a region, such as amount of rainfall, wind status and insecticide application are particularly important factors in vector biology. In the Black Sea region, yearly averages of temperature, humidity and rain are reported as 13.0 °C (4.2–22.1 °C), 71%, 842.6mm^3^, respectively. When compared, these values are lower than the Western Anatolia region of Turkey where the disease is seen frequently, reportedly 16.3 °C (6.4–26.8 °C), 63%, 2, 725.9mm^3^ respectively. These data are important factors affecting vector abundance. Hence the existence of *Culicoides* species in the Black Sea region was far less than the western and southern part of Turkey (14).

## Conclusion

Mosquitoes and midges have got a crucial role in the transmission of arboviruses. The risk of occurrence for the investigated arboviruses will continue depending upon many factors including (i) the presence of these viruses in Turkey and its neighbouring countries (ii) uncontrolled livestock movements (iii) global warming and climate changes. A large scale project, inclusive of both people and animals, should be planned. In addition, effective arboviral disease surveillance systems that can detect outbreaks of emerging zoonotic and non-zoonotic infection should be developed.

## References

[1] Liang G Gao X Gould EA (2015) Factors responsible for the emergence of arboviruses; strategies, challengers and limitations for their control. Emerg Microbes Infect. 4(3): e18. 26038768 10.1038/emi.2015.18 PMC4395659

[2] Jupille H Vega-Rua A Rougeon F Failloux AB (2014) Arboviruses: variations on an ancient theme. Future Virol. 9(8): 733–751.

[3] Adams MJ Lefkowitz EJ King AMQ Harrach B Harrison RL Knowles NJ Kropinski AM Krupovic M Kuhn JH Mushegian AR Nibert M Sabanadzovic S Sanfaçon H Siddell SG Simmonds P Varsani A Zerbini FM Gorbalenya AE Davison AJ (2017) Changes to taxonomy and the International Code of Virus Classification and Nomenclature ratified by the International Committee on Taxonomy of Viruses (2017). Arch Virol. 162 (8): 2505–2538. 28434098 10.1007/s00705-017-3358-5

[4] Alatoom A Payne D (2009) An overview of arboviruses and bunyaviruses. Lab Med. 40(4): 237–240.

[5] Adelman ZN Miller DM Myles KM (2013) Bed bugs and infectious disease: a case for the arboviruses. PLoS Pathog. 9: e1003462. 23966852 10.1371/journal.ppat.1003462 PMC3744395

[6] Karabatsos N (1985) International Catalogue of Arthropod-Borne Viruses. 3rd ed San Antonio Am Soc Trop Med Hyg.

[7] Ergunay K Gunay F Oter K Erisoz-Kasap O Orsten S Akkutay AZ Erdem H Ozkul A Alten B (2013) Arboviral surveillance of field-collected mosquitoes reveals circulation of West Nile virus lineage 1 strains in eastern Thrace, Turkey. Vector Borne Zoonotic Dis. 13(10): 744–752. 23919608 10.1089/vbz.2012.1288

[8] Wilson AJ Mellor P (2009) Bluetongue in Europe: past, present and future. Phil Trans R Soc B. 364: 2669–2681. 19687037 10.1098/rstb.2009.0091 PMC2865089

[9] Ozkul A Yildirim Y Pinar D Akcali A Yilmaz V Colak D (2006) Serological evidence of West Nile virus (WNV) in mammalian species in Turkey. Epidemiol Infect. 134(4): 826–829. 16316496 10.1017/S0950268805005492 PMC2870448

[10] Burgu I Urman HK Akca Y Yonguc AD Mellor PS Hamblin C (1992) Serologic survey and vector surveillance for bluetongue in southern Turkey. In: Bluetongue, African Horse Sickness and Related Orbivirusus. Walton BI Osburn TE (Eds), CRC Press Inc, Boca Raton, Flo pp. 168–174.

[11] Temizel EM Yesilbağ K Senturk S Maan SA Mertens-Clement PP Batmaz H (2009) Epizootic hemorrhagic disease in cattle, western Turkey. Emerg Infect Dis. 15(2): 317–319. 19193283 10.3201/eid1502.080572 PMC2662652

[12] Yildirim Y Gokce G Kirmizigul AH Erkilic EE Yilmaz V Tan MT Ozgunluk I (2015) Molecular and serological investigation of Akabane virus infection in cattle in Kars-Turkey. Isr J Vet Med. 70(3): 52–57.

[13] Campbell JA Pelham-Clinton CA (1960) A taxonomic review of the British species of *Culicoides* Latreille (Diptera: Ceratopogonidae). Proc R Soc Edinburgh [Biol.]. 67(3): 181–302.

[14] Dik B Kurt M Aydin I (2008) Karadeniz Bölgesi *Culicoides* (Diptera: Ceratopogonidae) Türleri Üzerine Bir Araştırma. Bornova Vet Kont Araş Enst Derg. 30: 23–26 [In Turkish].

[15] Lanciotti RS Kerst AJ Nasci RS Godsey MS Mitchell CJ Savage HM Komar N Panella NA Allen BC Volpe KE Davis BS Roehrig JT (2000) Rapid detection of West Nile virus from human clinical specimens, field-collected mosquitoes, and avian samples by a TaqMan Reverse Transcriptase-PCR Assay. J Clin Microbiol. 38(11): 4066–4071. 11060069 10.1128/jcm.38.11.4066-4071.2000 PMC87542

[16] Shaw AE Monaghan P Alpar HO Anthony S Darpel KE Batten CA Guercio A Alimena G Vitale M Bankowska K Carpenter S Jones H Oura CA King DP Elliott H Mellor PS Mertens PP (2007) Development and initial evaluation of a real-time RT-PCR assay to detect blue tongue virus genome segment 1. J Virol Methods. 145(2): 115–126. 17586061 10.1016/j.jviromet.2007.05.014

[17] Stram Y Kuznetzova L Guini M Rogel A Meirom R Chai D Yadin H Brenner J (2004) Detection and quantitation of akabane and aino viruses by multiplex real-time reverse-transcriptase PCR. J Virol Methods. 116(2): 147–154. 14738981 10.1016/j.jviromet.2003.11.010

[18] Wilson WC O’Hearn ES Tellgren-Roth C Stallknecht DE Mead DG Mecham JO (2009) Detection of all eight seroytpes of Epizootic hemorrhagic disease virus by realt-time reverse transcription polymerase chain reaction. J Vet Diagn Invest. 21(2): 220–225. 19286501 10.1177/104063870902100207

[19] Aziz-Boaron O Klausner Z Hasoksuz M Shenkar J Gafni O Gelman B David D Klement E (2012) Circulation of bovine ephemeral fever in the Middle East--strong evidence for transmission by winds and animal transport. Vet Microbiol. 158(3–4): 300–307. 22445538 10.1016/j.vetmic.2012.03.003

[20] Elbers ARW Koenraadt CJM Meiswinkel R (2015) Mosquitos and *Culicoides* biting midges: vector range and the influence of climate change. Rev Sci Tech Off Int Epiz. 34(1): 123–137. 10.20506/rst.34.1.2349 26470453

[21] Ozer N Ergünay K Simsek F Kaynas S Alten B Caglar SS Ustacelebi S (2007) West Nile virus studies in the Sanliurfa Province of Turkey. J Vector Ecol. 32 (2): 202–206. 18260509 10.3376/1081-1710(2007)32[202:wnvsit]2.0.co;2

[22] Kilpatrick AM Kramer LD Campbell SR Alleyne EO Dobson AP Dazsak P (2005) West Nile virus risk assessment and the bridge vector paradigm. Emerg Infect Dis. 11(3): 425–429. 15757558 10.3201/eid1103.040364 PMC3298247

[23] Walker PJ Klement E (2015) Epidemiology and control of bovine ephemeral fever. Vet Res. 46: 124. 26511615 10.1186/s13567-015-0262-4 PMC4624662

[24] Jennings M Mellor P (1989) *Culicoides*: biological vectors of Akabane virus. Vet Microbiol. 21(2): 125–131. 2609498 10.1016/0378-1135(89)90024-2

[25] Albayrak H Ozan E (2010) Molecular detection of avian influenza virus but not West Nile virus in wild birds in northern Turkey. Zoonoses Public Health. 57(7–8): e71–e75. 20298488 10.1111/j.1863-2378.2010.01327.x

[26] Yazici Z Albayrak H Ozan E Gumusova S (2012) The first investigation of West Nile Virus in horses using real time RT-PCR in Middle Black Sea Region. J Arthropod Borne Dis. 6(2): 151–155. 23378973 PMC3547302

[27] Albayrak H Ozan E (2010) Orta Karadeniz Bölgesinde Ruminant ve Tek Tırnaklılarda Kan Emici Sineklerle Nakledilen Bazı Arboviral Enfeksiyonların Seroprevalansı. Kafkas Univ Vet Fak Derg. 16(1): 33–36.

[28] Albayrak H Ozan E (2013) Seroepidemiological study of West Nile virus and Rift Valley fever virus in some of mammalian species (Herbivores) in northern Turkey. J Arthropod Borne Dis. 7: 90–93. 23785699 PMC3684502

[29] Ertürk A Tatar N Kabakli O Incoglu S Cizme SG Barut FM (2004) The current sitiuation of blutongue virus in Turkey. Vet Ital. 40(3): 137–40. 20419651

[30] Gumusova SO Yazici Z Albayrak H (2006) Orta Karadeniz Bölgesinde koyunlarda Mavidil Virus Enfeksiyonunun Serolojik Olarak [Inodot]ncelenmesi. Vet Hek Mikrob Derg. 6(1–2): 9–11.

[31] Oguzoglu TC Erturk A Cizmeci SG Koc BT Akca Y (2015) A report on bovine ephemeral fever virus in Turkey: antigenic variations of different strains of EFV in the 1985 and 2012 outbreaks using partial glycoprotein gene sequences transbound. Transbound Emerg Dis. 62(5): e66–e70. 24219124 10.1111/tbed.12187

[32] Tonbak S Berber E Yoruk MD Azkur AK Pestil Z Bulut H (2013) A large-scale outbreak of bovine ephemeral fever in Turkey, 2012 J Vet Med Sci. 75(11): 1511–1514. 23800972 10.1292/jvms.13-0085 PMC3942977

